# Deconstruction of physical fitness assessment system and medical rehabilitation countermeasures for physically disabled teenagers with natural language processing technology

**DOI:** 10.3389/fpubh.2022.964030

**Published:** 2022-08-04

**Authors:** Dan Wang, Pu Sun

**Affiliations:** ^1^College of Physical Education and Sports, Beijing Normal University, Beijing, China; ^2^College of Physical Education, Luoyang Normal University, Luoyang, Henan, China

**Keywords:** physically disabled adolescents, physical fitness, medical rehabilitation, natural language processing technology, physical fitness assessment system

## Abstract

Disability is a component of social relations and the existence of social reality. An effective solution to the problem of persons with disabilities is a measure of human civilization and social progress. The strength of the implementation of disability policy is the key to the realization of the original intention of “effectively solving the problem of disabled persons and promoting their development”. The purpose of this paper is to study the physical fitness evaluation system and medical rehabilitation strategies of physically handicapped adolescents with natural language processing technology. It mainly adopts literature research, interview and comparative analysis. Taking the rehabilitation policy for the disabled as the research starting point, the current situation of mental health of physically disabled adolescents will be examined, and the influence of physical exercise attitude and exercise level on mental health will be explored. And by comparing the difference in physical exercise level and mental health of physically handicapped youth and able-bodied youth, the effect of physical exercise on mental health of physically handicapped youth is further explained. This paper selects a total of 760 physically disabled students and able-bodied students from a secondary school as subjects, and uses research methods such as questionnaire survey and computer test to investigate the current situation of mental health of physically disabled adolescents and the relationship between physical exercise and mental health. The experimental results show that the spatiotemporal judgment experiment uses pixel difference as an indicator, and the judgment error of limb residual limbs is significantly larger than that of healthy limbs (*p* < 0.01). In the span of spatial location memory, the grades of the limb-restrained students were significantly lower than that of the able-bodied students (*p* < 0.05).

## Introduction

Natural language processing is widely used in today's development environment. Especially in the past 20 years, with the development of the Internet, there is a strong demand for this technology, and its text processing analysis in various fields has practical application significance. Physically disabled people lack the ability to engage in certain activities due to partial functional loss or abnormality, and therefore, have to take care of others. In daily life, people with physical disabilities not only have many physical and psychological problems themselves, but also cause great pressure to their caregivers.

Numerous studies have shown that physical exercise can promote people's mental health. However, studies on the relationship between physical activity and mental health in persons with disabilities are relatively rare. China is at an important stage of building a harmonious socialist society, and the mental health of the disabled is a key issue that needs our attention. The results of the Second National Sampling Survey of Disabled Persons show that among the disabled population in China, there are 24.12 million people with physical disabilities, ranking first in the total number of disabled people of all types. Therefore, the mental health of the physically handicapped has become a top priority, so the investigation of their mental health is of great significance. The innovation of this paper lies in the use of natural language processing technology to study the physical fitness evaluation system and medical rehabilitation countermeasures of physically handicapped adolescents, which has certain innovation and practicability.

## Related work

Due to the natural growth of the population, other social and environmental problems and the arrival of an aging society, more and more scholars have conducted research on the physical health of people with disabilities. The purpose of the Wieczorek M study was to determine the attitudes of adolescents toward persons with intellectual disabilities and the changes in their attitudes through participation in purposeful educational activities (1). Kilin F N aimed to identify obesity, stunting, and nutritional habits in children and adolescents with disabilities (2). Fegert J M was taking action to implement a social code for all children with disabilities. A structured and scientific assessment of individual barriers to participation as well as academic performance was required (3). Khazova S A analyzed the relationship between figures of internal defects, personality traits and self-attitudes of disabled adolescents (4). However, the shortcomings of these studies are that the detection accuracy and effectiveness are not high.

With the development of economy and technology, the application of natural language processing technology is becoming more and more important, and many scholars have carried out research on it. Bao Y tried to use natural language processing technology to predict hot news and help operators to write articles about hot news (5). Khader M studied a big data sentiment analysis method, applying several language and natural language processing (NLP) preprocessing techniques on the Twitter dataset to study their impact on big data classification accuracy (6). Dou J acquired a large amount of ICH data and utilized natural language processing (NLP) techniques to extract domain knowledge from ICH text data (7). To effectively diagnose diabetes, Abokhzam AA proposed a method based on ML grid search algorithm (8). However, the shortcoming of these studies is the uncertainty of data quality, and the calculation and analysis of massive data is very complicated, so the research data aspects still need to be improved.

## Methods of natural language processing technology

### Physical disability

#### Definition of physical disability

Human's understanding and definition of disability has gone through a long historical process, from the initial individual disability to “social disability” (9). Initially, people believed that the difficulties faced by disabled people were caused by physical disabilities, so the social work for disabled people focused on helping disabled people adapt to their special conditions after disability. Slowly, people discovered that the real cause of the plight of the disabled is not the disability itself, or that the disability cannot be the root cause. It is because the defects of social organization and management cannot provide sufficient conditions for the disabled, which leads to their plight and makes disability a problem. Physical disability refers to the loss of limbs or paralysis or deformity of limbs and trunk, resulting in different degrees of functional loss or dysfunction of the human motor system.

#### The role of physical exercise for the physically handicapped

**Figure 1:**
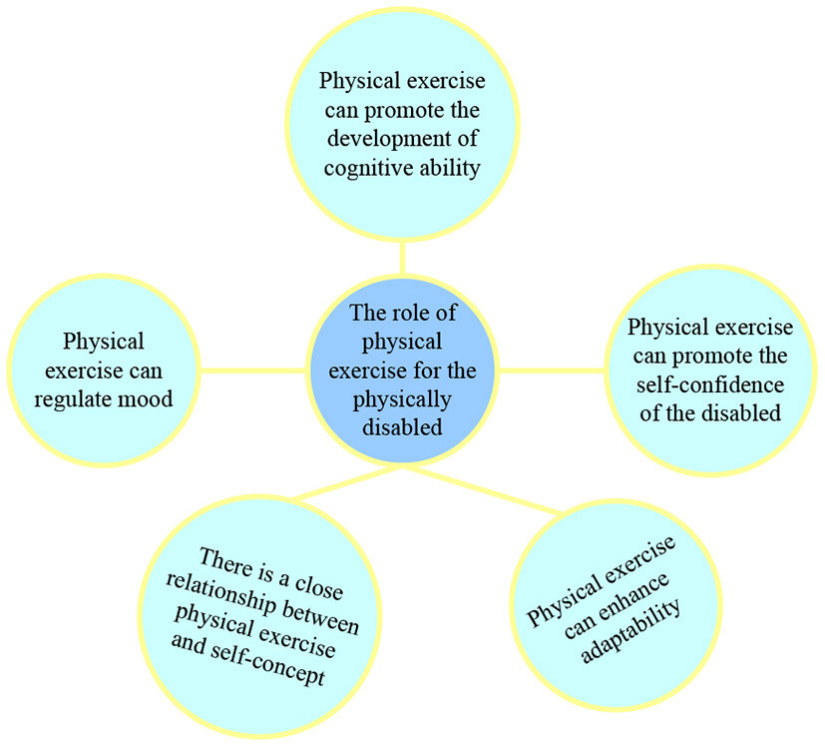
The role of physical exercise for the physically handicapped.

Scientific and reasonable physical exercise and physical exercise can help us enhance our physical fitness, reduce the incidence of diseases, and promote physical health. According to the thought of mind-body interaction theory, in recent years, the function of “healthy mind” of physical exercise has been gradually recognized by everyone, and has gradually attracted the attention of researchers (10). Several studies have shown that physical activity can not only improve people's physical health, but also promote mental health (Figure 1).

### Natural language processing technology

**Figure 2:**
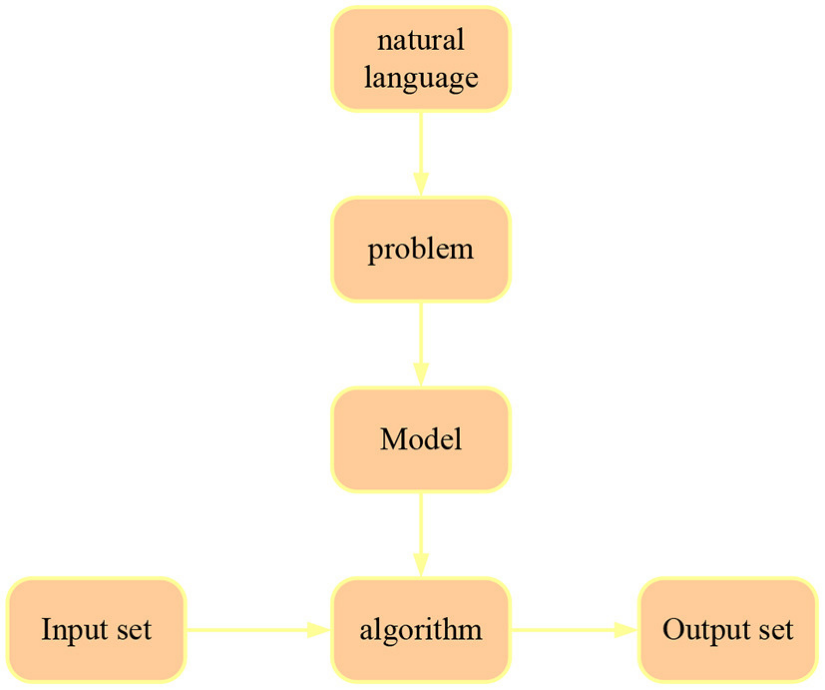
Natural language processing model.

Natural language processing technology is to use electronic computers to analyze and process language units at all levels of natural language (such as characters, words, sentences, paragraphs, chapters, etc.) (11). The process of natural language processing is the process of abstracting a specific problem of natural language based on the input set and output set to build a model, and designing an effective algorithm related to this problem according to this model. The process is shown in Figure 2.

The natural language processing process is divided into several stages: Lexical analysis is the first and most basic of the whole process, including dividing words into word strings and tagging words with parts of speech. Syntactic analysis is to clarify the structure of the sentence based on the results of the previous step: semantic analysis is to understand the internal meaning of the input based on the above preparations. Discourse analysis and pragmatic analysis focus on the influence of paragraphs and even the environment on the meaning of sentences. Because in daily life, our understanding of natural language is carried out in stages, but several stages are parallelized at the same time, so it is awkward to process the above natural language in stages. But in fact, under the circumstance that parallel processing cannot be realized at present, computer processing of natural language can only be carried out in such a staged form. The main algorithms involved in each stage of processing are introduced below (12).

The earliest application of natural language understanding technology in education is to detect grammatical errors. With the development of technology, natural language understanding has more application scenarios in teaching. Natural language understanding will bring new ways of learning to learners in areas such as machine translation, machine understanding, and question answering systems. It can be found that natural language understanding will bring new ways of learning for learners in fields such as machine text analysis and question answering systems. Some researchers have summarized the application scenarios of natural language understanding in the field of education into four aspects: (1) Text analysis and knowledge management, such as machine correction, machine translation, etc.; (2) Natural interactive interface of artificial systems, such as speech recognition and synthesis systems; (3) Application of corpus in educational tools, such as corpus and its retrieval tools; (4) Applied research in language teaching, such as educational games for language learning.

Machine text analysis: Traditionally, for the judgment of subjective questions, such as discussion, composition, etc., machine review cannot give effective feedback. With the gradual maturity of natural language understanding technology, automatic review of open-ended questions can be achieved by relying on artificial intelligence technology. The most successful application is Juku English composition correction. Machine review helps students to obtain timely feedback when they practice independently, which greatly improves the efficiency and effect of learning (13).

Question and answer system: At present, many application products have appeared in the question and answer system, such as Siri of Apple mobile phone, Cortana of Microsoft, Xiaodu of Baidu intelligent robot, etc. After they receive text or voice messages, they interpret the content and then automatically respond with relevant responses. In teaching, the question answering system can act as a virtual assistant to solve students' personalized problems, answering and tutoring students' questions in a natural interactive way. Watson, a virtual teaching assistant developed by IBM, can answer students' questions by establishing a library of experts in the field of education.

#### Support vector machine theory

Support Vector Machine can be regarded as a neural network with hidden layers, explaining SVM from the perspective of neural network (14). Support vector machine is based on the VC dimension theory of statistical theory and the principle of minimum structural risk. It can better solve practical problems such as small samples, non-linearity, high dimensionality and local minima, and is especially suitable for pattern recognition and classification.

##### Optimal classification hyperplane

The so-called optimal classification hyperplane means that the classification surface can not only separate the two classes correctly, make the training error rate 0, but also maximize the classification interval. The optimal hyperplane classification function is calculated as follows:

When the samples are linearly separable, the optimal hyperplane: ω • *X*+*C* = 0 normalizes it so that for linearly separable sample sets:


(1) $$\begin{array}{l} \left(X_{1},Y_{1}\right),\left(X_{2},Y_{2}\right),\cdots \;,\left(X_{n},Y_{n}\right),X_{u}\in R^{n};Y_{u}\in \\ \left\{-1,1\right\}\left(u=1,2,\cdots \;,n\right) \end{array}$$

Satisfy:


(2) $$\begin{array}{l} Y_{u}\left[\left(\omega •A_{u}+C\right)\right]\geq 1\left(u=1,2,\cdots \;,n\right) \end{array}$$

That is:


(3) $$\begin{array}{l} \{\begin{array}{l} \min T\left(r\right)=\frac{1}{2}{||r||}^{2}\\ Y_{u}\left[\left(\omega •X_{u}\right)+C\right]\geq 1\left(u=1,2,\cdots \;,n\right) \end{array} \end{array}$$

At this time, the classification interval is $\frac{2}{\left||\omega |\right|}$, and making the interval the largest is equivalent to making ||ω||^2^ the smallest. The training sample points satisfying Equation 3 are support vectors, and the optimal problem of this inequality constraint is solved in its Lagrange multiplier space. The constraint problem in Formula 3 is transformed into its dual form:


(4) $$\begin{array}{l} \{\begin{array}{l} \max\left(\overset{n}{\underset{u=1}{\sum }}\alpha_{u}-\frac{1}{2}\overset{n}{\underset{u,v=1}{\sum }}\alpha_{u}\alpha_{v}B_{u}B_{v}\left(X_{u}•X_{v}\right)\right)\\ \overset{n}{\underset{u=1}{\sum }}\alpha_{u}Y_{u}=0,\alpha_{u}\geq 0 \end{array} \end{array}$$

After solving the above problem:


(5) $$\begin{array}{l} g\left(X\right)=sgn\left[\overset{n}{\underset{u=1}{\sum }}\alpha_{u}Y_{u}\left(X_{u}•X_{v}\right)+C\right] \end{array}$$

The parameter C is the penalty coefficient, which is used to balance the maximum interval and the minimum classification error, and its choice has a great influence on the performance of the support vector machine.

**Figure 3:**
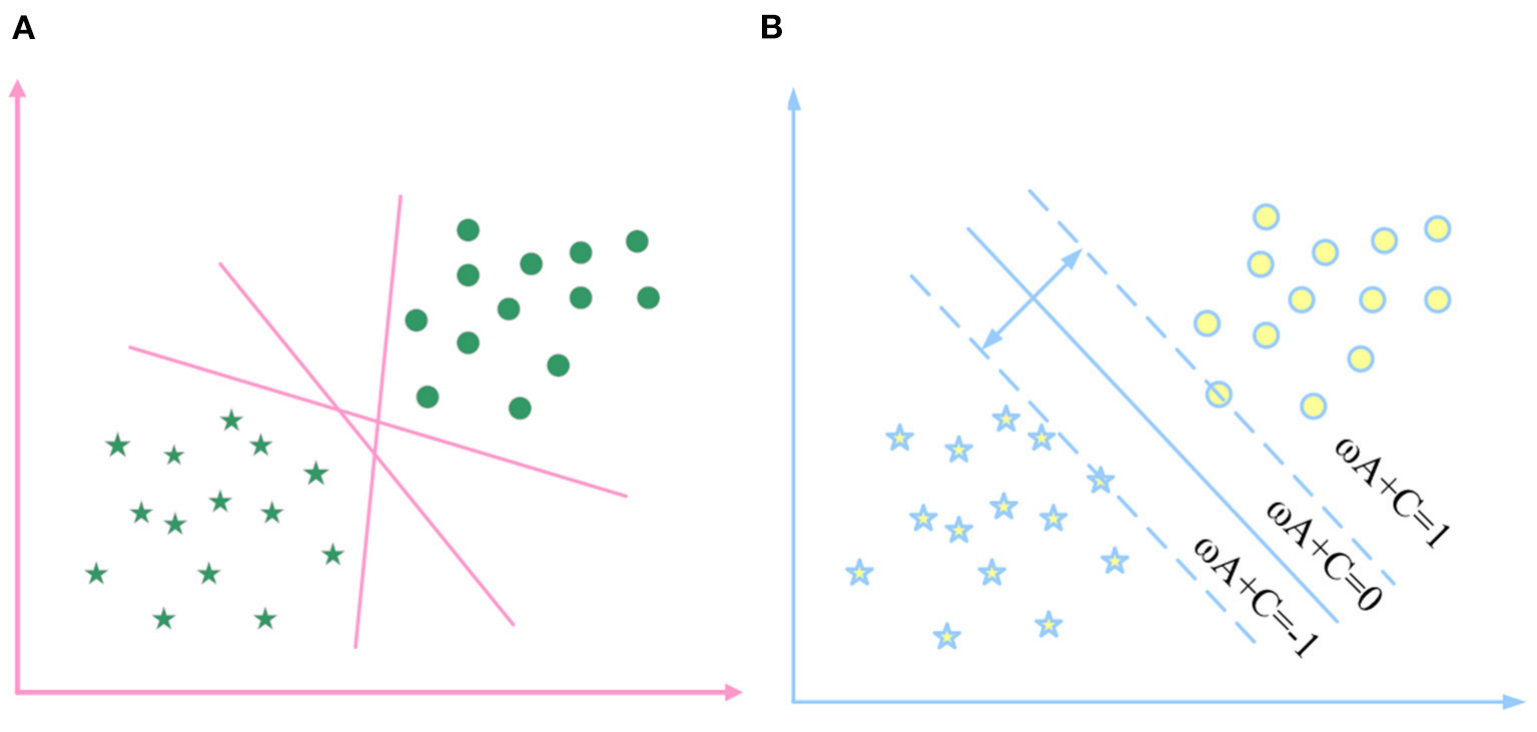
The optimal classification hyperplane in the linearly separable case.

In Figure 3, straight line ω*X*+*C* = 0 is the optimal classification line, ω*X*+*C* = 1 and ω*X*+*C* = −1 are respectively the straight lines that are the samples closest to the optimal classification line and are parallel to the classification line. The training sample points on it are the support vector machine, the distance between ω*X*+*C* = 1 and ω*X*+*C* = −1 is the maximum classification interval, and the size is $\frac{2}{\left||\omega |\right|}$.

In the case of linearly separable samples, the optimal hyperplane classification figure is shown in Figure 3.

When the sample is linearly inseparable, a non-negative slack variable is added to Formula 2 to account for ξ_*u*_ ≥ 0, namely:


(6) $$\begin{array}{l} Y_{u}\left[\left(\omega •X_{u}\right)+C\right]\geq 1-\xi_{u},\left(u=1,2,\cdots \;,n\right) \end{array}$$

Then the objective function becomes:


(7) $$\begin{array}{l} \min T\left(r,\xi \right)=\frac{1}{2}||r{||}^{2}+D\left(\overset{n}{\underset{u=1}{\sum }}\xi_{u}\right) \end{array}$$

The constraints become:


(8) $$\begin{array}{l} \overset{n}{\underset{u=1}{\sum }}\alpha_{u}Y_{u}=0,0\leq \alpha_{u}\leq D,u=1,2,\cdots \;,n \end{array}$$

That is:


(9) $$\begin{array}{l} \{\begin{array}{c} \max\left(\overset{n}{\underset{u=1}{\sum }}\alpha_{u}-\frac{1}{2}\overset{n}{\underset{u,v=1}{\sum }}\alpha_{u}\alpha_{v}Y_{u}Y_{v}\left(X_{u}•X_{v}\right)\right)\\ \overset{n}{\underset{u=1}{\sum }}\alpha_{u}Y_{u}=0,0\leq \alpha_{u}\leq D,u=1,2,\cdots \;,n \end{array} \end{array}$$

##### Support vector machine

The basic idea of the support vector machine method is to map the input data to a high-dimensional inner product space through a non-linear mapping, and perform linear classification in this high-dimensional feature space. At the same time, all necessary computations are made in the input space by using the kernel function.

Support vector machines can solve high-dimensional and non-linear classification problems, minimize structural risk, and take into account training errors and generalization problems. It has a good ability to solve small sample data sets and has great advantages in the field of pattern recognition.

**Figure 4:**
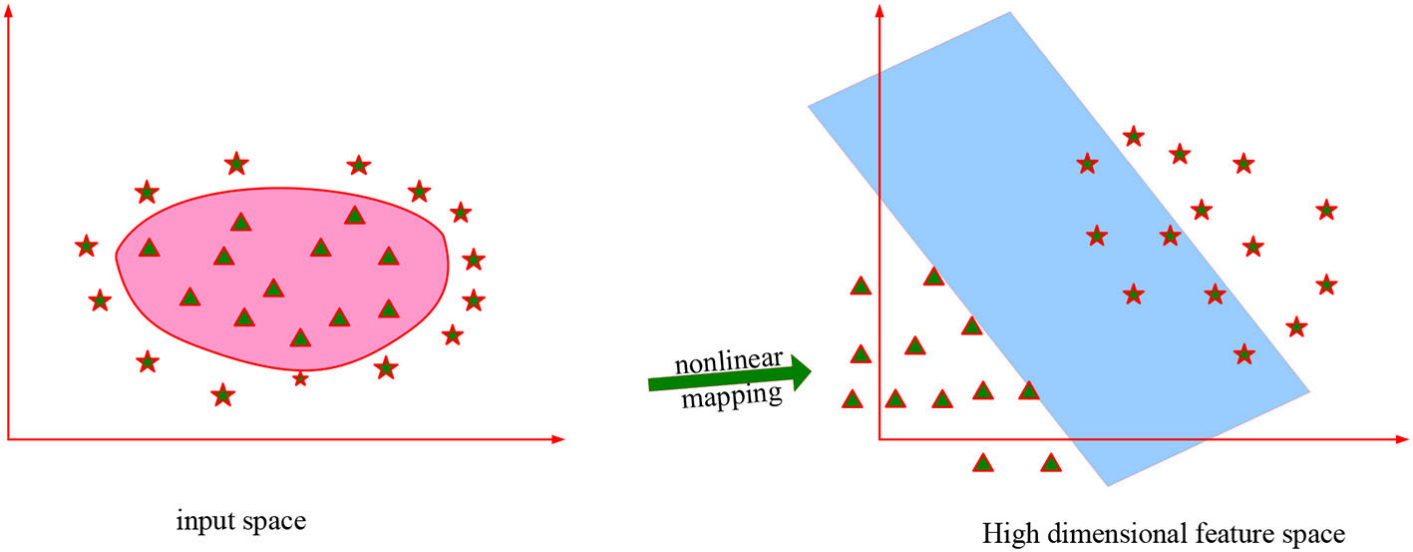
Support vector machine space map.

The support vector machine can understand the hyperplane division of the prepared information in the high-level highlight space, and maintain a strategic distance from the non-linear surface division calculation in the first information space. And gave a strategy to deal with the “curse of dimensionality” problem caused by computing (15). As shown in Figure 4:

The description work under the SVM dataset system has the following characteristics: It is a direct combination of a series of non-linear capabilities, with the help vector as the limit. Along these lines, the declaration of grouping capability is only related to the number of help vectors and the autonomy of the room components.

#### Improved algorithm based on K-means algorithm

1) Implementation principle of K-Means clustering algorithm

K-means is an iterative distance-based algorithm. Its goal is to distribute n given data instances into k clusters, hence the name K-means. In this way, each observation instance is less distant from the center point of its cluster than the center point of other clusters. The k-means algorithm first randomly divides the input points into k initialized groups, or uses certain rules to group them. Then calculate the center point of these groups of data, secondly regroup the data, and divide some data close to the center point together. The above process is repeated, and the data center is calculated repeatedly until the position of the data center does not change, that is, the convergence is stopped (16).

However, in actual research work, to solve a problem, people cannot directly obtain perfect theoretical results. People have to use theoretical inspiration to take the first step. That is to solve the problem approximately first, and then improve it step by step to get closer to perfection. Through the above analysis, it is known that it is an NP-hard problem to obtain a global optimal solution. However, if multiple local optimal solutions can be obtained, and then the optimal solution among these local optimal solutions can be regarded as the final result by a certain method of evaluating the quality of clustering results, such a result can be regarded as an objective clustering result (17).

There are three mainstream methods for the calculation of cluster center points:

2) The value of Minkowski Distance method 1 can be taken at will, which can be positive, negative, or infinity.

(10) $$\begin{array}{l} d_{uv}=\sqrt[{\lambda }]{\overset{n}{\underset{k=1}{\sum }}{\left|a_{uk}-a_{vk}\right|}^{\lambda }} \end{array}$$

3) Euclidean Distance method one is the case of λ = 2 in the first formula.

(11) $$\begin{array}{l} d_{uv}=\sqrt[{\;}]{\overset{n}{\underset{k=1}{\sum }}{\left|a_{uk}-a_{vk}\right|}^{2}} \end{array}$$

4) CityBlock Distance method one is the case of λ = 1 in the first formula.

(12) $$\begin{array}{l} d_{uv}=\overset{n}{\underset{k=1}{\sum }}|a_{uk}-a_{vk}| \end{array}$$

**Figure 5:**
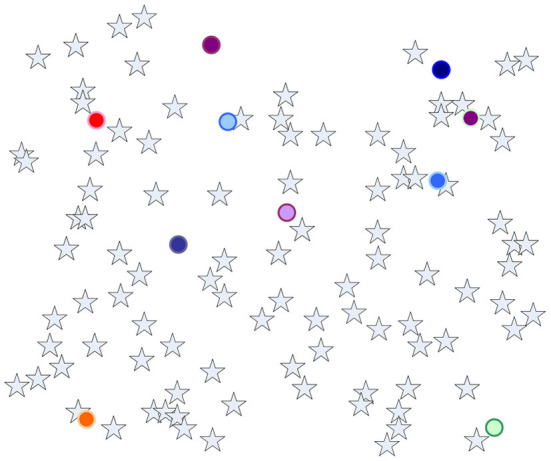
An example of the K-means clustering algorithm.

**Figure 6:**
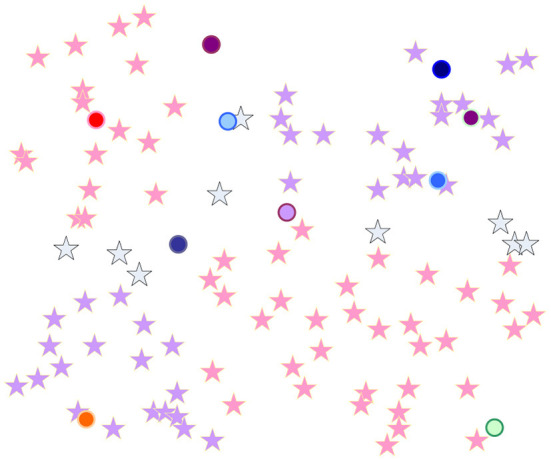
Divide 100 instances into nearest clusters.

**Figure 7:**
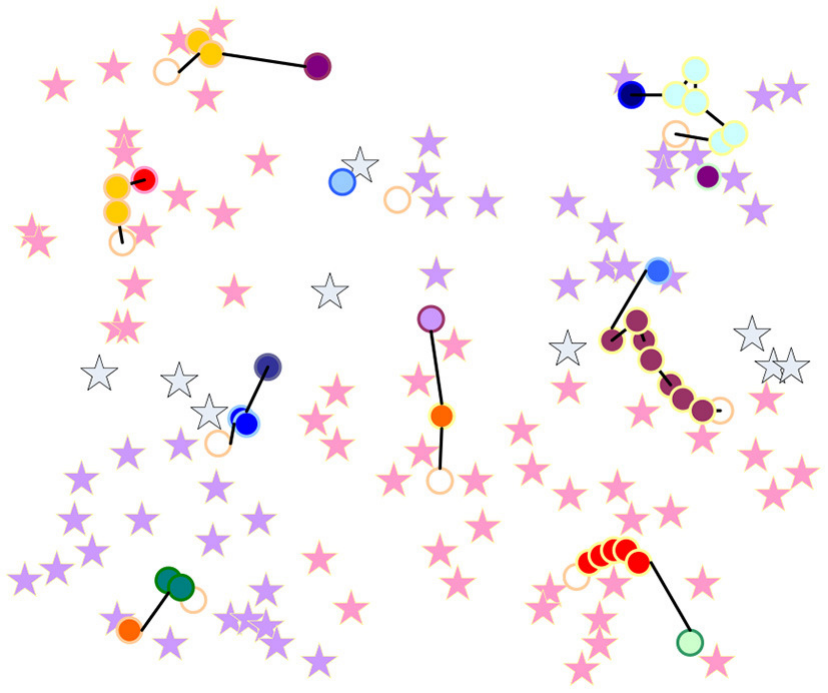
Change process of cluster centers.

An example of the K-means clustering algorithm is listed below, and its object is 100 instances with two-dimensional features (18). Convergence after 10 iterations. Suppose k = 10. The initial distribution of 100 observation points is shown in Figure 5. The whole process is shown in Figures 6, 7. The small rectangles in the figure are ten randomly selected center points:

So, how to evaluate the quality of a clustering result? We take the sum of the squares of the distance between each data observation point and the center point of their corresponding category as the evaluation index, and use it to construct an evaluation function evaluate(C). The smaller the value of evaluate(C), the better the clustering effect (19).

#### BP algorithm (backpropagation pass)

The training algorithms adopted by classical neural networks such as CNN are all BP algorithms. The BP algorithm calculates the error value between the current output of the network and our expected output, and lets the error propagate back to adjust the network weights until the error between the output and our expected value remains stable within a reasonable range, and the training ends (20). The rate of change of the error is obtained by derivation, as follows:


(13) $$\begin{array}{l} \frac{\partial E}{\partial y}=\frac{\partial E}{\partial p}\frac{\partial p}{\partial y}=\delta \end{array}$$

Among them, E is the error function. Since $\frac{\partial p}{\partial y}=1$, then $\frac{\partial E}{\partial y}=\frac{\partial E}{\partial p}=\delta$, this derivative is the key to making the error generated by the upper layer pass down layer by layer. Backpropagation applies the following formula:


(14) $$\begin{array}{l} \delta^{q}={\left(R^{q+1}\right)}^{T}\delta^{q+1}\circ g^{\prime }\left(p^{q}\right) \end{array}$$

Operation ∘ represents the cross product operation, that is, each element is multiplied, and the sensitivity of the output layer neurons is different from each other:


(15) $$\begin{array}{l} \delta^{q}=g^{\prime }\left(p^{q}\right)\circ \left(b^{n}-t^{n}\right) \end{array}$$

Finally, the rule of δ is applied to each neuron to update the weights. Specifically, applying δ of each neuron to scale its input, which can be described in terms of a vector, is for layer 1. For a given layer, the cross product between its input value and its sensitivity is equivalent to the partial derivative of the error value to its weights connected to each neuron. Thus, the product of the partial derivative and a negative learning rate is finally updated as the weight of the neurons in this layer:


(16) $$\begin{array}{lll} \frac{\partial E}{\partial R^{q}} & = & a^{q-1}{\left(\delta^{q}\right)}^{T} \end{array}$$

(17) $$\begin{array}{lll} \Delta R^{q} & = & -\eta \frac{\partial E}{\partial R^{q}} \end{array}$$

The update method for the bias parameter is basically the same, and in fact, each update of the weight *R*_*ij*_ corresponds to a specific learning rate η_*ij*_.

## Experiment and deconstruction of physical fitness assessment and rehabilitation of physically handicapped adolescents

### Destructuring objects and methods

#### Questionnaire survey method

**Figure 8:**
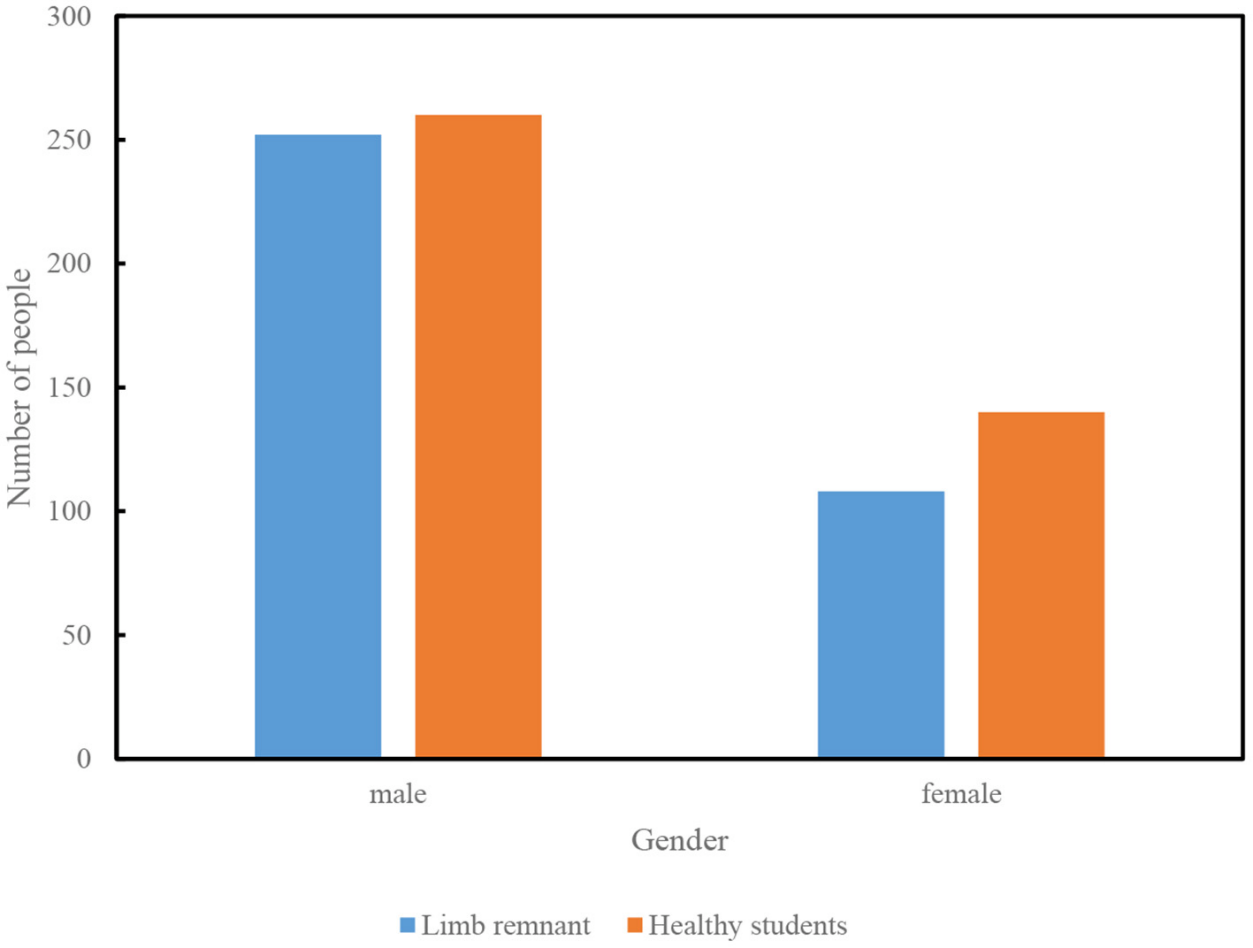
Study subjects.

Select students with physical disabilities and able-bodied students in a secondary school as subjects (21, 22). A total of 800 test papers were distributed, and the recovery rate was 96.6%. Excluding 40 invalid questionnaires, 760 valid questionnaires were obtained, with an effective rate of 95%. The reliability, validity and objectivity of the questionnaires were tested before the questionnaires were distributed. Among them, 252 males and 108 females were selected for the disabled students, totaling 360; the healthy students were selected as 260 males and 140 females, totaling 400 (Figure 8).

#### Expert interview method

According to the research content, senior experts were consulted and interviewed, and the issues related to this research were discussed and exchanged deeply.

#### Mathematical statistics

Using SPSS 11.0 statistical software, statistical analysis was carried out on the data related to the rolls.

### Relationship between exercise attitude, exercise behavior and mental health of the disabled

#### Deconstruction of the attitude of the disabled toward physical exercise

It is not difficult to see from the analysis of the survey results that 34.83% of the respondents are those who like and like it very much, and only 1.12% who do not like it. The number of people who think it is necessary to engage in physical exercise accounted for 45.17% of the respondents, unnecessary accounts for only 2.47%. This is enough to show that the disabled have a certain experience and understanding of the significance of physical exercise, and physical exercise has occupied an important position in the minds of the disabled. At the same time, it also reflects the disabled people's demand for sports and their desire for life, hoping to improve their physical quality and lifestyle through physical exercise (see Table 1 for details).

**Table 1:** Statistical list of attitudes of disabled persons toward physical exercise.

**Category**	**n**	**%**
Like it very much	41	11.23
Like	85	23.60
Commonly	44	12.13
Dislike	5	1.12
Be necessary	163	45.17
Indifferent	16	4.27
Unnecessary	6	2.47

#### Deconstruction of disabled persons' participation in physical exercise programs

The survey results show that: at present, the sports that Chinese disabled people like and often participate in include walking, running, table tennis, badminton, basketball and so on. These projects have strong fitness, practicality and fun, and are relatively safe and easy to participate in. However, it is also found that the physical exercise of the disabled has a single way, mainly focusing on the above-mentioned types of projects. How to further develop some sports that disabled people love is an important part of developing sports for disabled people.

I think it can be considered from the following: the public lacks understanding of the sports activities that the disabled participate in, the public thinks that many sports activities are not suitable for the disabled, and the disabled themselves also lack bold attempts and experience of some sports. On the one hand, it may be due to the limited conditions of local economic development and geographical environment. On the other hand, it also shows that the relevant departments still have serious deficiencies in promoting and publicizing the existing sports programs suitable for the disabled and in the development, transformation and utilization of new sports programs (see Table 2 for details).

**Table 2:** Statistics list of physical exercise items selected by disabled persons.

**Project**	**n**	**%**
Running, walking	114	31.46
Table tennis	36	10
Basketball	37	10.11
Volleyball	26	6.97
Football	64	17.53
Swimming	27	7.42
Aerobics	45	12.36
Other	11	4.16

#### Factors affecting the exercise of disabled persons

As shown in Table 3, the factors hindering the physical fitness of the disabled are multi-faceted and multi-angle, and the lack of venues and equipment provided by the community accounts for the highest proportion. The existence of psychological barriers not only affects their health, but also hinders their confidence and motivation to exercise. Carrying out a psychological counseling office or holding a mental health lecture can eliminate the psychological problems of the disabled and help them embark on the correct path of physical fitness. Some disabled people believe that physical inconvenience is also one of the reasons that hinder disabled people from exercising. Physiological obstacles make it impossible to complete even some basic life movements, let alone exercise. At present, most of the fitness programs and facilities of Jianjin people in Huzhou City are not suitable for disabled people to participate, which is one of the reasons why disabled people are unwilling or uninterested to participate in fitness exercises.

**Table 3:** Factors influencing exercise for people with disabilities.

**Factors that hinder exercise**	***n***	**%**	**Factors that promote exercise**	***n***	**%**
Psychological disorders	64	17.53	entertainment	36	10
Economic obstacles	84	23.15	recovery	97	26.85
Physiological disorders	73	20.22	Disease prevention	42	11.46
Insufficient equipment in the community	46	12.58	build up a good physique and improve one's health	98	26.97
Backward cultural knowledge	7	1.80	take part	9	2.47
Lack of sports instructors in the community	39	10.56	boost self-esteem	24	6.63
Lack of fitness awareness	19	5.17	social communication	26	7.19
Afraid of being laughed at by others	19	5.17	other	28	8.43
Other	9	3.82			

### Time deconstruction of physically disabled people participating in physical exercise

**Figure 9:**
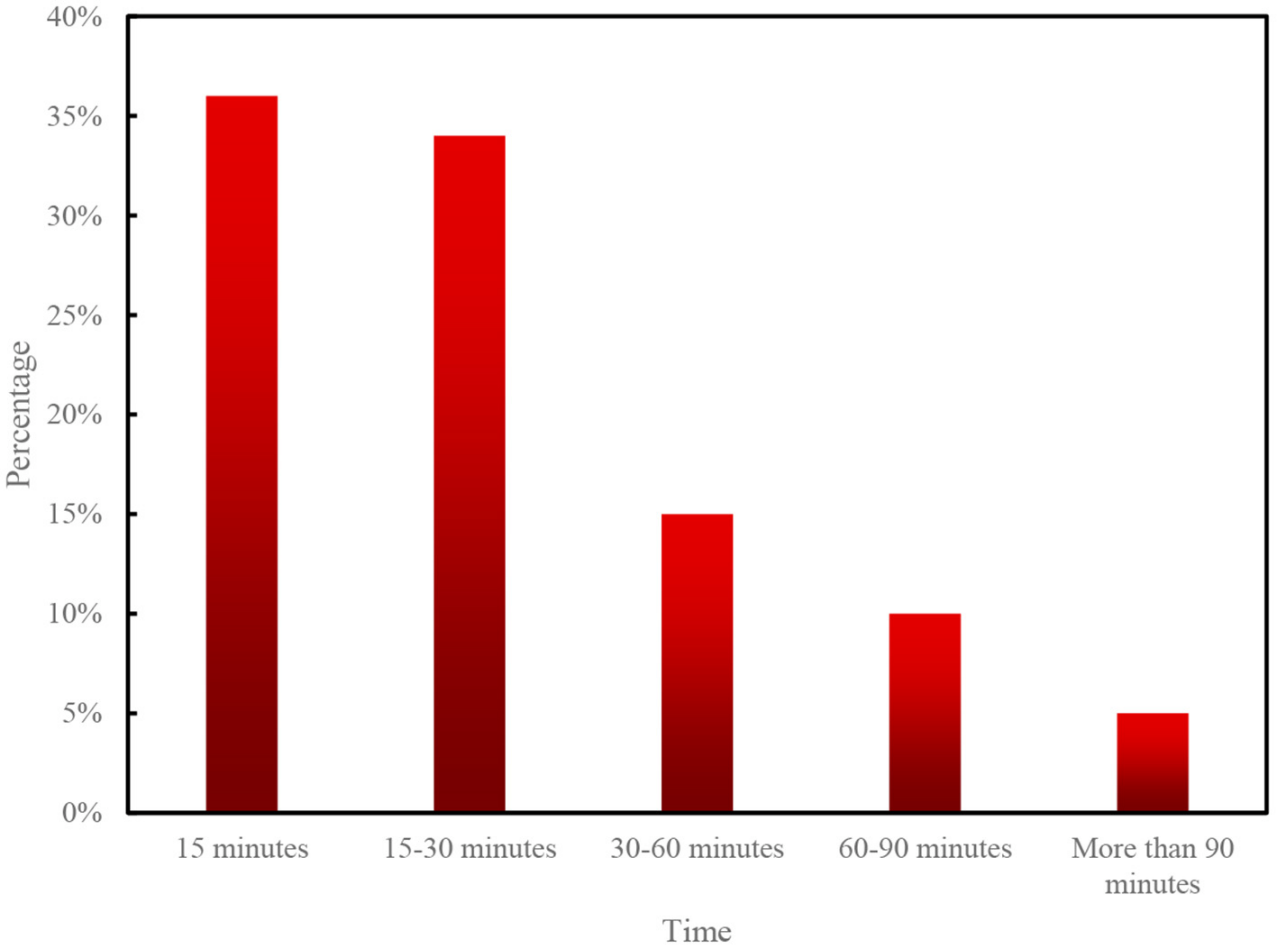
Legend of the percentage of physical exercise time for the physically handicapped.

The survey results show that: at present, the number of physically disabled people in China takes 15 min and 15–30 min for each activity, accounting for 36 and 34% of the number of people, respectively; 15% for 30–60 min; and 10% for 60–90 min; 5% for more than 90 min (see Figure 9 for details).

**Figure 10:**
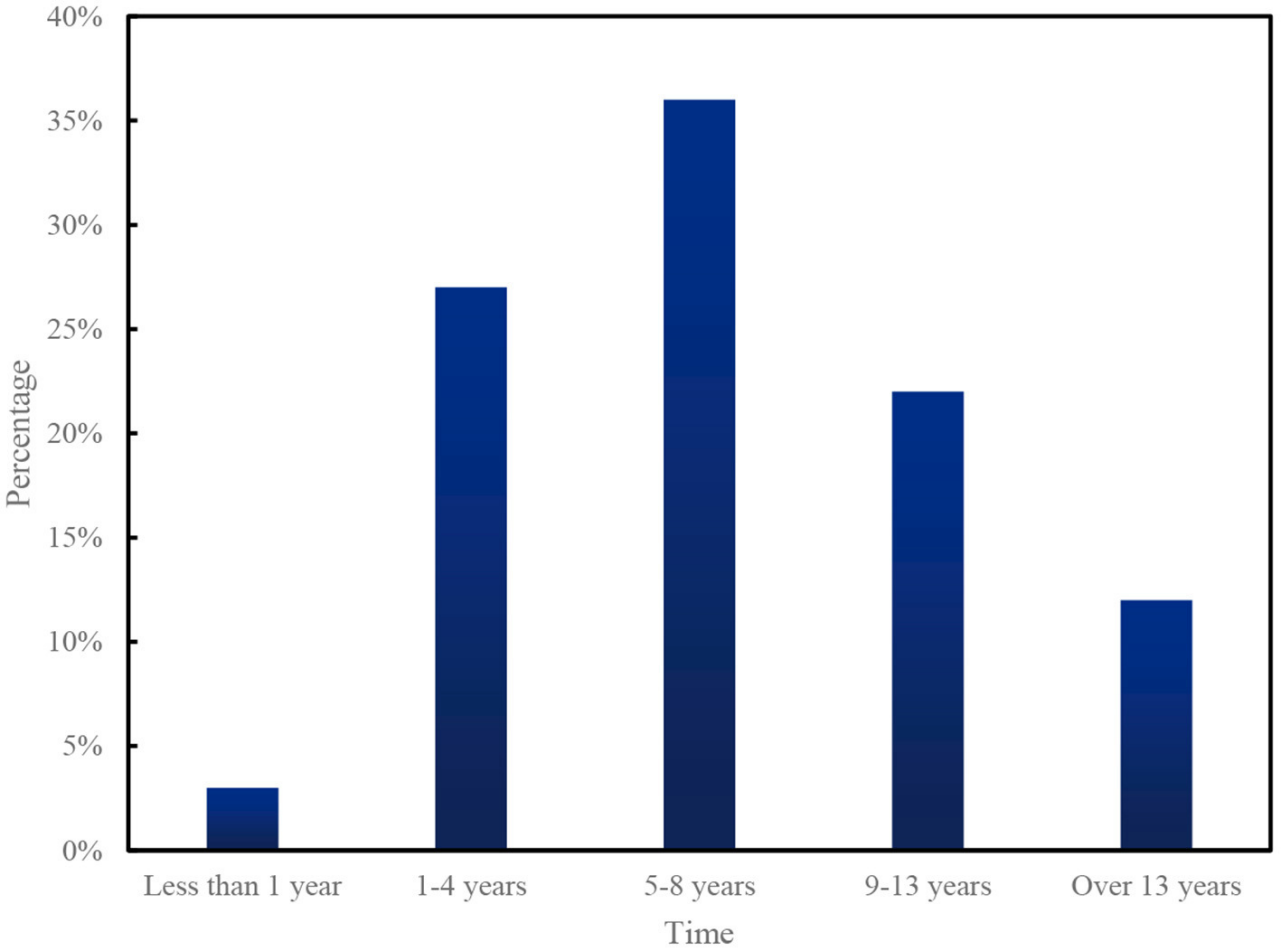
Legend of the percentage of physical training years for persons with physical disabilities.

The survey results show that: at present, the number of years of physical exercise for the physically disabled in China is 5–8 years, accounting for 36%, 1–4 years for 27%, 9–13 years for 22%, and 12% for more than 13 years. 3% for less than 1 year (see Figure 10 for details).

**Figure 11:**
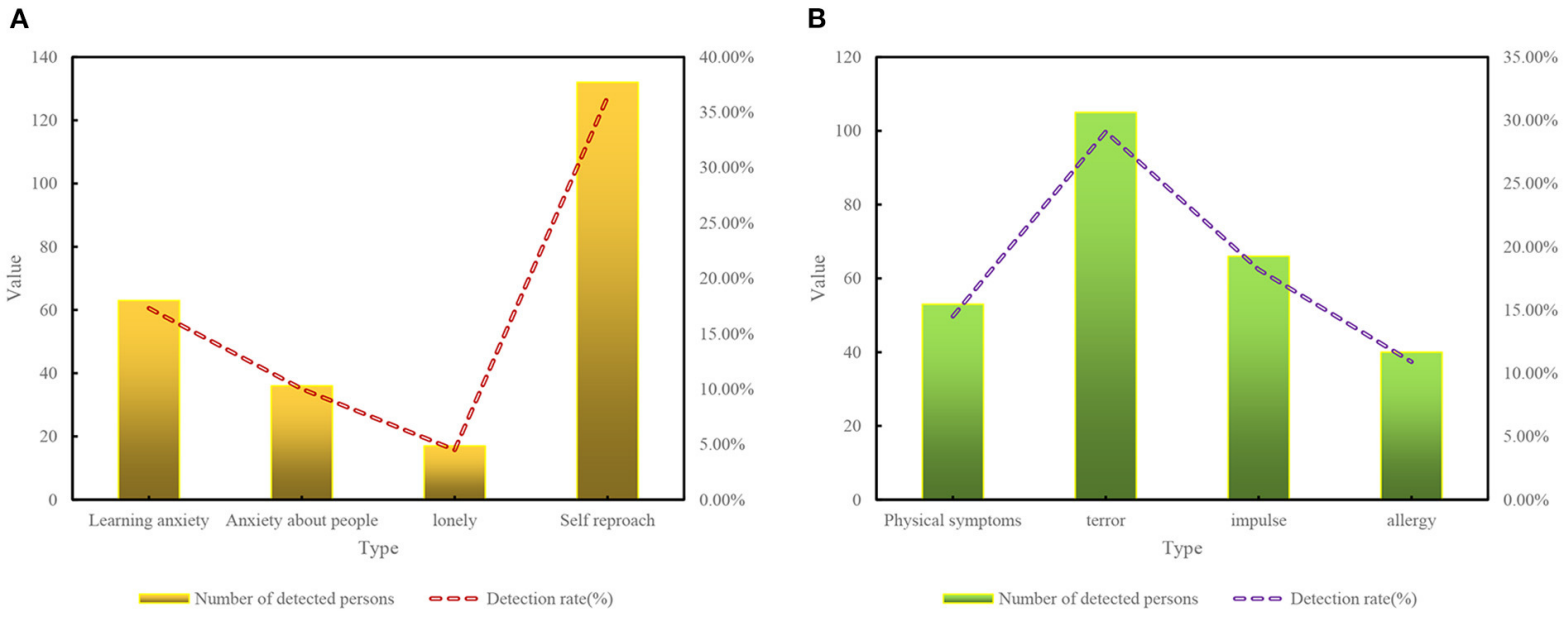
Mental health problem detection rate.

According to Figure 11, in general, 88 of the 400 able-bodied students have serious mental health problems, and the detection rate of psychological problems is 22%. The data shows that the overall mental health of able-bodied students is not optimistic. From the detection rate of each content scale, the detection rate of various psychological problems from high to low is (1) self-blame tendency, (2) terror tendency, (3) impulsive tendency, (4) learning anxiety, (5) physical symptoms, (6) allergic tendency, (7) anxiety tendency toward people, (8) loneliness tendency.

**Figure 12:**
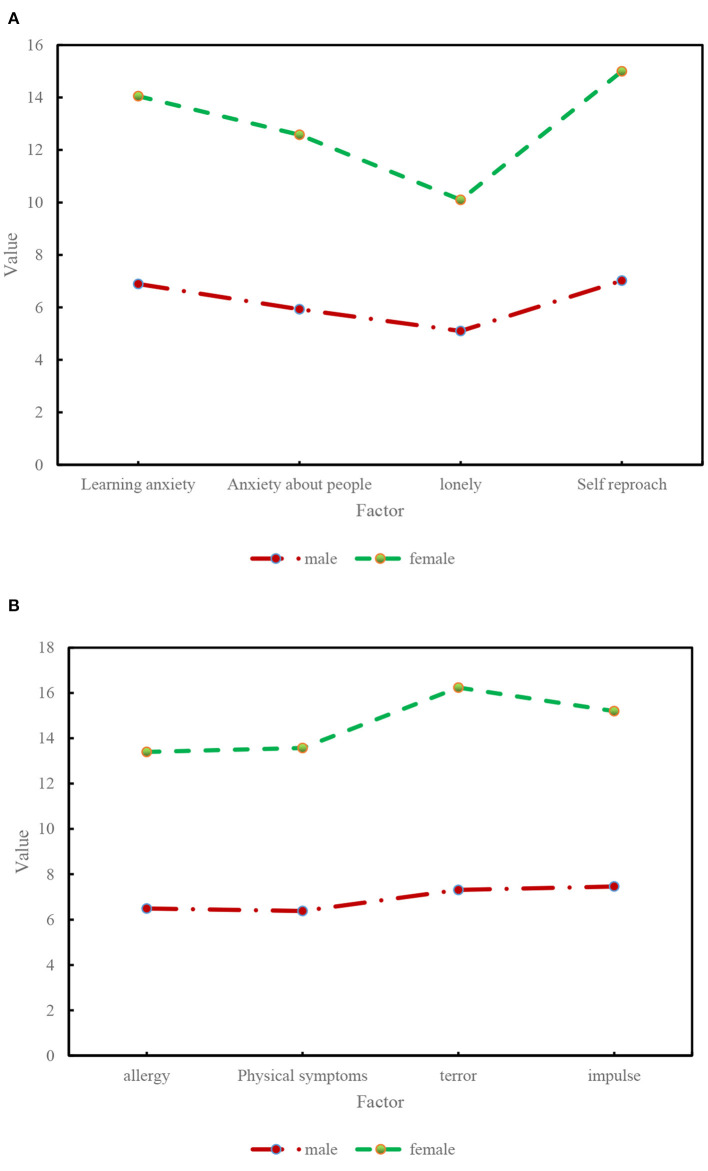
Differences in the mental health status of able-bodied students by gender.

The results in Figure 12 show that there is a significant difference in the gender dimension of the mental health score of able-bodied students, and the score of girls is significantly higher than that of boys. In the scores of each content scale, there are significant differences between male and female students in three factors: learning anxiety, self-blame tendency, and terror tendency. Specifically, girls scored significantly higher than boys on these three factors. There was no significant difference between male and female students in terms of anxiety, loneliness, allergy and other factors.

### Comparison of motor cognitive ability between disabled and able-bodied students

The results in Table 4 show that there are significant differences in the results of the five cognitive ability tests between the residual limb and the healthy photogenic response (hand), light response (foot), visual choice response time, spatiotemporal judgment, and spatial location memory span. Among them, the response time of light response (hand), light response (foot), and visual choice response time was used as an index, and the response time of limb remnants was significantly longer than that of healthy students (*p* < 0.01). In the spatio-temporal judgment experiment, the pixel difference was used as the index, and the judgment error of the limb residual limb was significantly larger than that of the healthy limb (*p* < 0.01). In the span of spatial location memory, the grades of the limb-restrained students were significantly lower than that of the able-bodied students (*p* < 0.05).

**Table 4:** Comparison of motor cognitive ability between disabled and able-bodied students.

**Test**	**Limb remnant**	**Healthy students**	**t**
Light reaction (hand)	235.89, 27.50	225.70, 27.44	2.656**
Photo reaction (foot)	301.96, 21.91	293.61, 20.253	2.734**
Visual selective reaction time	717.29, 165.54	617.85, 95.64	5.283***
Spatiotemporal judgment	28.82, 40.43	17.79, 14.06	2.667**
Spatial memory span	3.73, 0.79	4.01, 0.79	−2.533*

* indicates a small statistical difference, ** indicates a large statistical difference, and *** indicates a very large statistical difference.

## Conclusions

This study examined the mental health of adolescents with physical disabilities, although overall, students with physical disabilities were in the normal range of mental health levels. However, comparing the detection rate of this mental health problem with the results of other groups of studies found that: (1) Physical exercise level of physically disabled students is significantly lower than that of able-bodied students, and there is basically no significant difference between the two in exercise attitude. (2) The detection rate of mental problems in students with limb disabilities is higher than that in healthy students, and the psychological problems of learning anxiety are more obvious. (3) Both the able-bodied students and the physically handicapped students showed significant gender differences in the total score of mental health and the score of the content scale. The scores of girls on the three factors of learning anxiety, self-blame tendency and terror tendency are significantly higher than boys. (4) Physically disabled students are worse than healthy students in selected cognitive dimensions, and their cognitive ability development level is lower.

## Data availability statement

The original contributions presented in the study are included in the article/supplementary material, further inquiries can be directed to the corresponding author/s.

## Author contributions

All authors listed have made a substantial, direct, and intellectual contribution to the work and approved it for publication.

## Conflict of interest

The authors declare that the research was conducted in the absence of any commercial or financial relationships that could be construed as a potential conflict of interest.
